# Insights into antibiotic resistomes from metagenome-assembled genomes and gene catalogs of soil microbiota across environments

**DOI:** 10.7717/peerj.20348

**Published:** 2025-11-19

**Authors:** Xuemei Han, Huan Liu, Xue Bai, Diyan Li, Tao Wang, Hang Zhong, Yongfang Yao, Jing Sun

**Affiliations:** 1 College of Life Science, Sichuan Agricultural University, Yaan, China; 2 Department of Emergency, Ruijin Hospital, Shanghai Jiao Tong University School of Medicine, Shanghai, China; 3 School of Pharmacy, Chengdu University, Chengdu, China; 4 Chongqing Academy of Animal Sciences, National Center of Technology Innovation for Pigs, Chongqing, China

**Keywords:** Soil microbiome, Antibiotic resistome, Metagenome-assembled genomes, Regional variation

## Abstract

Antibiotic resistance poses a significant global health threat, and soil is recognized as a critical reservoir for antibiotic resistance genes (ARGs). To investigate soil microorganisms in the areas where both humans and common domestic animals (such as pigs and chickens) are present and active. In this study, we employed metagenomic sequencing to investigate the soil resistome across four Chinese provinces—Yunnan, Guizhou, Sichuan, and Jiangsu. From 111 soil samples, we generated metagenome-assembled genomes (MAGs) and gene catalogs to analyze microbial community composition, ARG distribution, and mobile genetic elements (MGEs). Our results revealed notable regional differences in microbial communities and ARG profiles. Pseudomonadota and Actinomycetota were the dominant phyla across samples, and ARG abundance was significantly higher in Sichuan, Yunnan, and Jiangsu compared to Guizhou. We also identified microbial taxa likely serving as ARG vectors, suggesting potential for horizontal gene transfer. Functional annotation indicated that metabolic functions, particularly carbohydrate and amino acid metabolism, were predominant, which may be associated with the composition of organic matter in the soil environment. Multidrug resistance genes are widespread in soil microbial communities and may spread through food chains or soil-water-plant systems, posing potential ecological and public health risks. MGEs showed significant regional variation and play a key role in the horizontal spread of ARGs. Together, these findings provide new insights into the soil antibiotic resistome and offer a foundation for developing targeted strategies to manage environmental antibiotic resistance.

## Introduction

Antibiotic resistance is an urgent global health threat, driven by the transfer of resistant bacteria and genes among humans, animals, and the environment (Larsson & Flach, 2022). Antimicrobial resistance (AMR) is estimated to cause around 700,000 deaths annually and could claim up to 20 million lives by 2050 if left unchecked (Abo Kamer et al., 2023; Darby et al., 2023). Soil is one of the most diverse ecosystems on Earth in terms of microbial diversity and is considered a major reservoir of ARGs and antibiotic-resistant bacteria (ARBs) (Liu et al., 2024; Van Goethem et al., 2018). These ARGs are increasingly linked to human health risks, while recent studies have also highlighted their close relationships with soil development processes and microbial community succession (Chen et al., 2022). The widespread exchange of resistance genes between environmental microbes and human-associated microbial communities increases the risk of colonization by resistant microbes (Hu, Gao & Zhu, 2017).

Metagenomics, the study of genetic material recovered directly from environmental samples (Wang et al., 2024), has revolutionized how we study microbial communities and their functions (Taş et al., 2021). Metagenome-assembled genomes (MAGs), constructed from short DNA reads, offer greater power in identifying microbial populations within complex environments (Almeida et al., 2019; Tyson et al., 2004). MAGs have expanded the genomic landscape of underrepresented habitats like soil (Anthony et al., 2024), providing new opportunities to study microbial ecology, function, and resistance gene dynamics (Nelkner et al., 2019). This approach enables a more detailed analysis of ARG diversity, abundance, potential hosts, and transmission mechanisms.

Previous research has underscored the role of soil microbiomes as a major gene pool for antibiotic resistance (Klümper et al., 2024). Environmental and agricultural factors contribute to regional differences in soil resistomes, with distinct microbial communities and ARG levels observed under varying conditions (Seyoum et al., 2021; Wang et al., 2020). Moreover, the sharp rise in infections caused by multidrug-resistant bacteria has driven urgent efforts to monitor environmental ARGs and assess their public health risks (Serwecińska, 2020). Most previous studies have focused on a single region or specific type of environment, lacking systematic comparative analyses across geographically and ecologically diverse areas. This is particularly true for regions where humans and common domestic animals coexist intensively and engage in frequent activities, such as areas that combine agricultural production with intensive livestock and poultry farming. Existing research has rarely systematically linked the metabolic pathways of soil microbial communities to ARG profiles. Nevertheless, microbial metabolic activities may affect the emergence and spread of ARGs by altering the composition of soil organic matter or microbial interaction networks.

This study focuses on the soil environments of four provinces in China: Yunnan, Guizhou, Sichuan, and Jiangsu. These regions not only span different geographical and climatic zones but also cover extensive areas with frequent human and domestic animal (*e.g.*, pigs, chickens) activities, providing conditions for exploring the regional variation of soil resistomes. By employing metagenomic sequencing technology, we obtained MAGs and a comprehensive gene catalog from 111 soil samples. The aim is to systematically analyze the composition of microbial communities, the distribution patterns of ARGs, the characteristics of mobile genetic elements, and microbial functional profiles. Through this approach, we seek to clarify the regional differences in soil resistomes, identify potential microbial carriers of ARGs, reveal the association between mobile genetic elements and the spread of ARGs. The aim is to provide new insights into the structure and dynamics of the soil resistome, and thereby supporting the development of more effective strategies to manage antibiotic resistance in soil environments and reduce associated human health risks.

## Materials & Methods

### Sample collection, DNA extraction, and sequencing

Soil samples were collected from four Chinese provinces: Sichuan (25), Yunnan (28), Guizhou (28), and Jiangsu (30). These regions were chosen to represent diverse geographic and ecological settings, including mountainous and plateau areas in the southwest and agricultural plains in the east. All four provinces are characterized by intensive human activities and livestock production, providing representative environments for studying human-animal-soil interactions. Sampling across these regions allowed the capture of ecological and agricultural variation relevant to soil microbial communities and ARGs. In total, 111 soil samples were obtained. With detailed metadata including location, altitude, and GPS coordinates recorded for each site (Table S1). At each sampling site, surface soil (0–1 cm depth) was collected according to a five-point sampling protocol using a sterile soil shovel. The five sub-samples obtained from each point were then combined to form a single composite sample. Subsequently, the composite soil was immediately passed through a 2-mm sieve to remove plant debris and gravel. Approximately 1–3 g of the composite soil was collected for each sample. To preserve sample integrity, all samples were frozen on dry ice after collection and subsequently stored at −80 °C. Total genomic DNA was extracted from soil samples using the Mag-Bind^®^ Soil DNA Kit (Omega Bio-tek, Norcross, GA, USA) following the manufacturer’s instructions. DNA concentration and purity were determined using a NanoDrop 2000 spectrophotometer, and integrity was assessed by 1% agarose gel electrophoresis.

DNA samples were fragmented to an average size of approximately 350 bp using a Covaris M220 instrument (Gene Company Limited, China) for the preparation of paired-end sequencing libraries. Library construction was carried out with the NEXTFLEX Rapid DNA-Seq Kit (Bio Scientific, Austin, TX, USA). Adapters comprising the full complement of sequencing primer binding sites were ligated to the blunt-ended DNA fragments. Paired-end sequencing was conducted on an Illumina NovaSeq 6000 platform (Illumina Inc., San Diego, CA, USA) at Majorbio Bio-Pharm Technology Co., Ltd. (Shanghai, China), utilizing the NovaSeq 6000 S4 Reagent Kit v1.5 (300 cycles) in accordance with the manufacturer’s protocols (https://www.illumina.com/).

### Metagenomic assembly

Quality control and sequence assembly were performed using the Majorbio Cloud Platform (http://www.majorbio.com). Adapter sequences and low-quality reads (length <50 bp or quality value <20) were removed using fastp (version 0.23.0) (Chen et al., 2018). Host-derived reads were removed by aligning against the human (GRCh38.p13), chicken (GRCg7b), and pig (Sscrofa11.1) genomes using BWA (http://bio-bwa.sourceforge.net, version 0.7.9a) (Li & Durbin, 2009). Clean reads were assembled using MEGAHIT (version 1.1.2) (Li et al., 2015).

### Metagenomic binning and MAG quality control

Three tools, Metabat2 (version 2.12.1) (Kang et al., 2015), MaxBin2 (version 2.2.5) (Alneberg et al., 2014), and CONCOCT (version 0.5.0) (Wu, Simmons & Singer, 2016), were used for binning. The resulting bins were consolidated using DAS Tools (version 1.1.0) (Sieber et al., 2018). RefineM (version 0.0.24) (Parks et al., 2017) was used to filter contigs based on genomic features such as GC content, tetranucleotide signatures, coverage, and taxonomy. MAG completeness and contamination were assessed with CheckM (version 1.0.12) (Parks et al., 2015) using lineage-specific marker genes. Only MAGs with ≥ 50% completeness and <10% contamination were retained (Parks et al., 2017).

Pairwise comparisons were performed using Mash (Ondov et al., 2016) and dereplication was conducted with dRep (version 3.4.2) (Olm et al., 2017), retaining the highest-quality MAG for each cluster at ≥ 99% average nucleotide identity (ANI). MAG coverage was calculated using CoverM (version 0.6.1). Taxonomy was assigned using Genome Taxonomy Database Toolkit (GTDB-Tk) (version 2.3.0) (Parks et al., 2018) based on 120 universal single-copy marker genes from the Genome Taxonomy Database (GTDB).

### Gene prediction and functional annotation

Gene prediction for all MAGs was performed using Prodigal (version 2.6.3) with the -p meta parameter (Hyatt et al., 2010). Only genes with a nucleic-acid length ≥ 100 bp were retained and subsequently translated into amino-acid sequences. The annotated protein sequences were automatically aligned against multiple databases using Diamond (Version 0.8.35, *E*-value ≤ 1e−5), including Kyoto Encyclopedia of Genes and Genomes (http://www.genome.jp/kegg/), COG (https://www.ncbi.nlm.nih.gov/research/cog-project/), CAZy (http://www.cazy.org/), CARD (https://card.mcmaster.ca/), and MGE (https://github.com/KatariinaParnanen/MobileGeneticElementDatabase).

### Data analysis

In this study, violin plots, clustered heatmaps, pie charts, bar plots, correlation scatter plots, and polar bar plots were generated using the Microbioinformatics platform (https://www.bioinformatics.com.cn). The MAG species Sankey diagram, correlation heatmap, and circular phylogenetic tree were generated using Python (v2.7.10). UpSet Venn diagrams, dual-matrix correlation heatmaps, KEGG histograms, COG annotation classification statistics, antibiotic resistance gene prediction classification statistics, and gene potential mobility analysis charts were produced using the Majorbio platform (http://www.majorbio.com). Additionally, UpSet Venn diagrams and interactive heatmap bar plots were generated with the assistance of the Paisainuo Gene Cloud platform (https://www.bioinformatics.com.cn).

To investigate the relationships between species distribution and environmental variables (including altitude, latitude, and longitude), as well as the associations between mobile genetic element MGE types and ARG categories, Spearman’s correlation coefficient was employed for analysis. Furthermore, Spearman’s rank correlation test was used to evaluate the correlation between the abundance of ARGs and MGEs. Spearman’s correlation calculation was employed, and significance was denoted by *p*-values, with * indicating *p* < 0.05, ** indicating *p* < 0.01, and *** indicating *p* < 0.001.

## Results

### Assembly of 1,136 microbial genomes from soil

**Figure 1:**
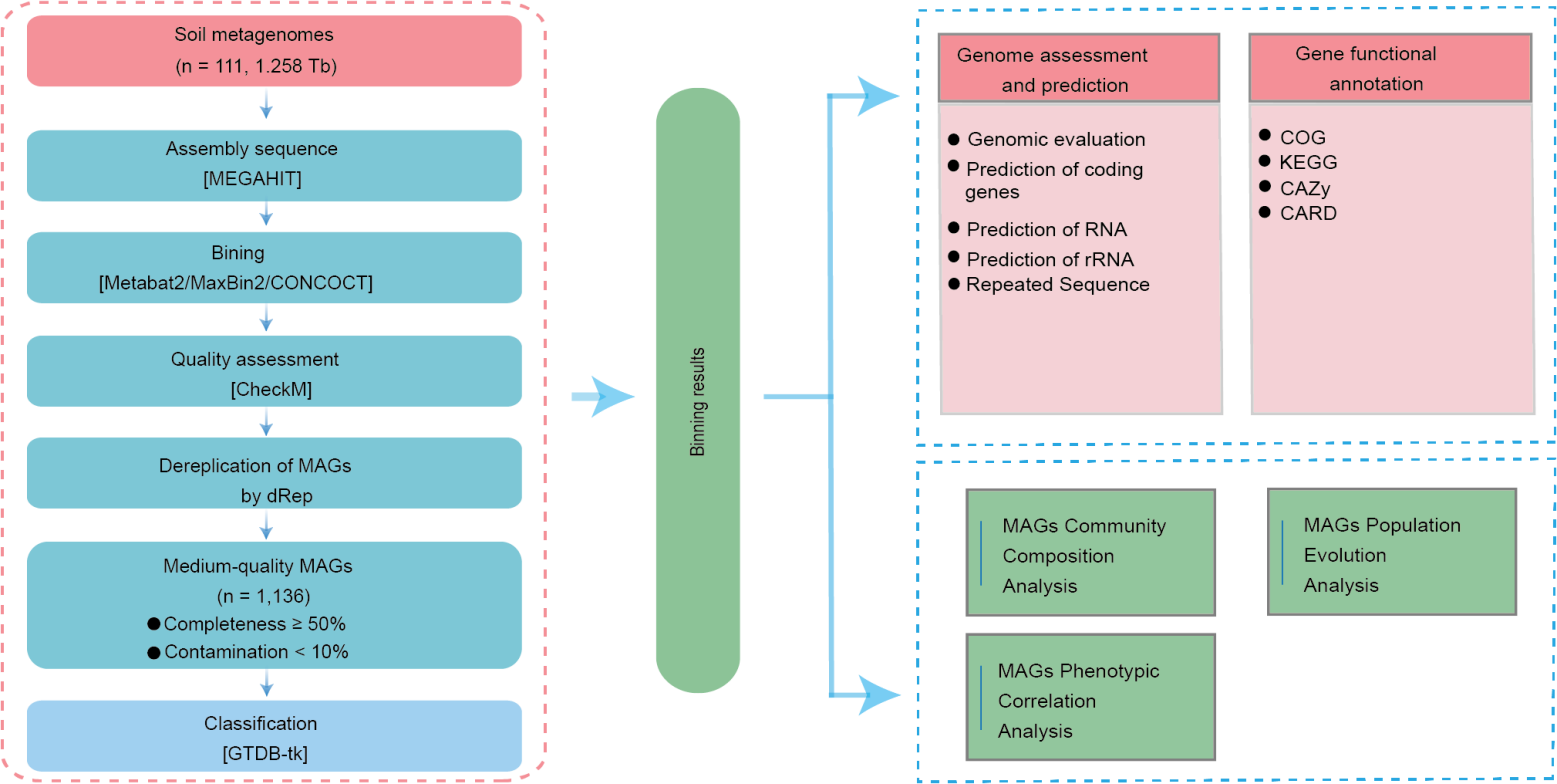
Overview of the assembled genomes and their functional annotations.

We constructed a metagenomic assembly gene catalog using sequencing data from 111 soil samples across four Chinese provinces (Fig. 1). High-throughput sequencing generated 1.276 Tb of raw data, averaging 11.50 Gb per sample. After stringent quality filtering, 1.258 Tb of high-quality data were retained, reflecting a 98.59% retention rate and an average effective sequencing depth of 11.33 Gb per sample (Table S1).

Dereplication at an average nucleotide identity (ANI) threshold of ≤ 99% and quality filtering yielded 1,136 MAGs that met our predefined standards for medium-quality assemblies (≥ 50% completeness and <10% contamination; Table S2). Among them, 176 MAGs met the MIMAG standard for high-quality MAGs defined by the Genome Standards Consortium (≥ 90% completeness and ≤ 5% contamination) (Bowers et al., 2017), while the remaining 960 were classified as medium-quality. The average completeness across all MAGs was 74.19%, with a mean contamination of 3.40% (Table S2).

To examine the genome characteristics of these soil-derived MAGs, we used metrics including total genome length, contig number, predicted coding sequences (CDS), GC content, N50, tRNA genes, rRNAs, and repetitive elements (Fig. S1A). The total genome length of all MAGs combined was 3.17 Gb. On average, each MAG contained 725 contigs, ranging from 1 to 3,135, indicating substantial variation in final assembly continuity among MAGs. We predicted 3,548,612 CDS in total, with a mean of 3,123 CDS per MAG, offering a rich resource for functional annotation.

The average GC content was 58.75%, ranging from 28.83% to 75.07%, reflecting the diversity characteristics of the soil microbial genomes. It is worth noting that the upper limit of 75.07% GC content comes from a *Cellulosimicrobium funkei* genome. Compared with the NCBI database, this value has reached the highest level of GC content reported for this species so far.

The overall average N50 was 12,271 bp. Notably, according to the MIMAG criteria (≥ 90% completeness and ≤ 5% contamination) (Bowers et al., 2017), the 176 high-quality MAGs also exhibited greater assembly continuity, with an N50 of up to 7,575,282 bp—considerably higher than the average of 6,364,488 bp observed for medium-quality MAGs (Table S3). In addition, we identified 36,249 tRNA genes, 684 rRNA genes, and 74,838 repetitive elements across the MAGs (Fig. S1A; Tables S3–S5).

### Microbial classification and composition

To classify the 1,136 MAGs, we aligned their sequences with the GTDB. The taxonomic distribution improved at higher ranks: 1,115 MAGs were assigned to a class, 1,047 to an order, 891 to a family, 223 to a genus, and 144 to a species (Table S6). All MAGs were taxonomically annotated using GTDB (Fig. S1B), which revealed 23 bacterial phyla (1,103 MAGs) and three archaeal phyla (33 MAGs). The most abundant bacterial groups included Actinomycetota (324 MAGs), Pseudomonadota (252 MAGs), and Bacteroidota (119 MAGs). Among archaeal MAGs, 20 out of 33 were unclassified at the species level. These archaeal genomes were distributed across Thermoproteota (31 MAGs), Thermoplasmatota (1 MAG), and Methanobacteriota (1 MAG). The average relative abundance of archaeal phyla is approximately 7.12% (Table S6).

**Figure 2:**
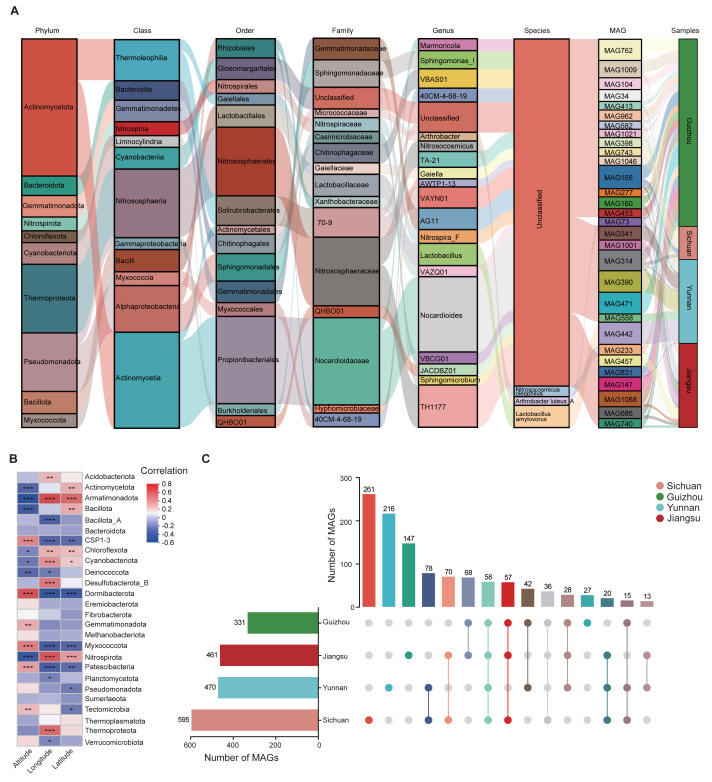
Composition and taxonomic characteristics of the soil MAGs community. (A) Sankey diagram showing the relative abundance of the top 30 most abundant MAGs across taxonomic ranks. Columns represent different taxonomic levels; colored bands indicate species, with length proportional to abundance. The colored bands connecting the pillars represent the correspondence between species/samples at different taxonomic levels from different regions. (B) Spearman correlation heatmap showing relationships between environmental factors (*x*-axis) and phylum-level MAG classifications (*y*-axis). Asterisks indicate significance levels (* *p* < 0.05, ** *p* < 0.01, ***p < 0.001). (C) Distribution of MAGs across provinces. Vertical bars represent the number of MAGs in shared or unique groups; horizontal bars indicate total MAG counts per province.

Using MAG-based Sankey diagrams, we analyzed species abundance across samples at multiple taxonomic levels, visualizing patterns of community structure and regional distribution. At the phylum level, Actinomycetota dominated the top 30 most abundant MAGs. As the taxonomic resolution increased to genus and species, the Sankey diagram displayed more complex branching. Some species showed regional enrichment; for instance, MAG442 (*Lactobacillus amylovorus*) was significantly enriched in Yunnan Province (Fig. 2A). Additionally, correlations were observed between MAGs abundance and environmental variables such as altitude, latitude, and longitude. For example, Dormibacterota abundance was positively correlated with altitude, while Armatimonadota abundance was positively associated with both latitude and longitude (Fig. 2B).

Further analysis revealed patterns of microbial community composition across provinces. Among the four regions, 58 MAGs were shared, including those from Pseudomonadota (18 MAGs), Actinomycetota (17 MAGs), Thermoproteota (14 MAGs), Cyanobacteriota (6 MAGs), Chloroflexota (2 MAGs), and Nitrospirota (1 MAG) (Fig. S2). We also identified 651 unique MAGs: 261 from Sichuan, 216 from Yunnan, 27 from Guizhou, and 147 from Jiangsu (Fig. 2C). Bacterial taxa found in more than 90% of samples from a region were defined as core bacteria. In Guizhou, five phyla met this threshold, indicating their wide distribution and potential ecological importance (Table S7).

To explore regional differences, the top 10 MAGs with the highest relative abundance were selected from the shared and endemic MAGs in each province in this study. We compared the top 10 most abundant MAGs, both shared and region-specific, within each province. At the genus level, shared MAGs in Yunnan, Guizhou, and Jiangsu showed greater similarity compared to those from Sichuan. Genera such as *Micrococcus* (MAG1095), *Arthrobacter* (MAG845), and *VBCG01* (MAG104) were dominant in Yunnan, Guizhou, and Jiangsu, while *Chroococcidiopsis* (MAG929), *Arthrobacter* (MAG802), and *Paracoccus* (MAG292) were more abundant in Sichuan, indicating a distinct MAG composition in that region (Fig. S3).

Genus-level analysis of region-specific MAGs also demonstrated clear provincial differentiation. Dominant genera included *VAYN01*, *UBA4720*, and *JACDBZ01* in Sichuan; *o_Gloeomargaritales*; *g_Unclassified* in Guizhou; *Sphingomonas_I*, *40CM-4-68-19*, and *CADCTB01* in Yunnan; and *Sphingomicrobium* and *VAYN01* in Jiangsu (Table S6).

### Functional annotations of MAGs

**Figure 3:**
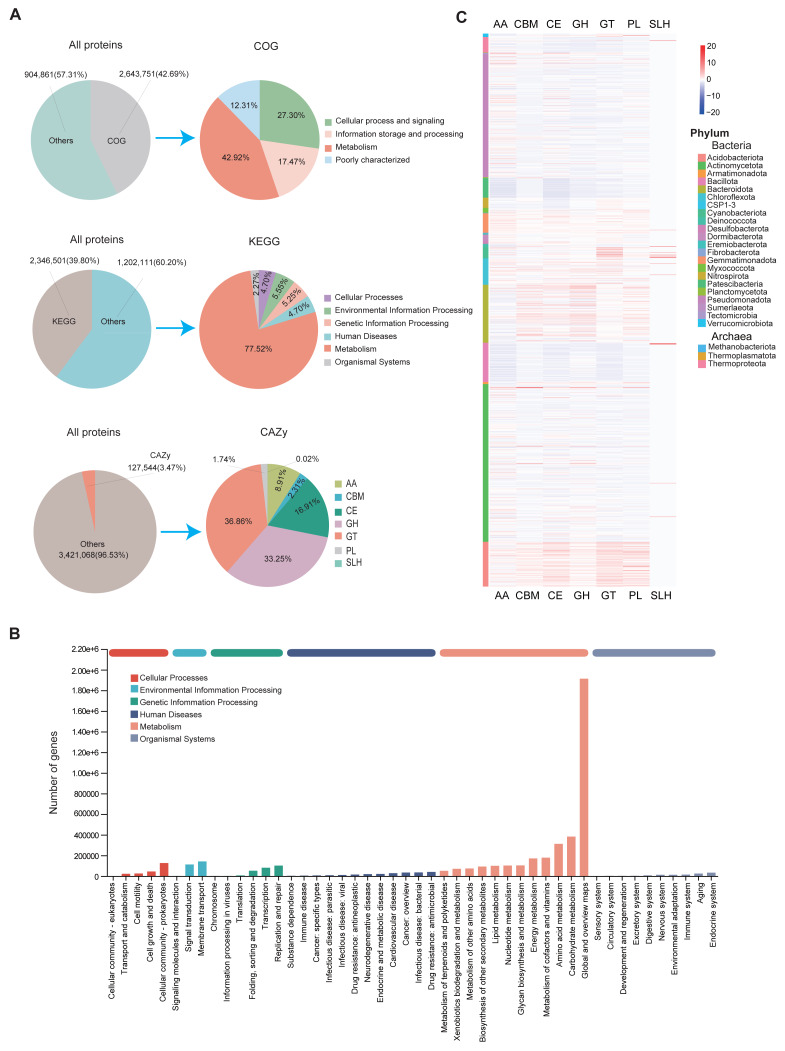
Functional annotation of MAGs. (A) Functional classification of predicted proteins from MAGs based on COG (upper), KEGG (middle), and CAZy (lower) databases. (B) KEGG pathway distribution by second-level category (*x*-axis). The *y*-axis shows the number of annotated genes. Bar colors indicate first-level classifications, and the top bar summarizes total gene counts per first-level category. (C) Heatmap of the distribution of seven CAZy categories across different microbial phyla.

The assembled MAGs were functionally annotated using three major databases: COG, KEGG, and CAZy. We found that 42.69% (2,643,751), 30.8% (2,346,501), and 3.47% (127,544) of the predicted proteins had at least one COG, KEGG, and CAZy function, respectively (Fig. 3A). According to the COG classification, the MAGs were categorized into four main groups: Cellular Processes and Signaling, Information Storage and Processing, Metabolism, and Poorly Characterized functions. These groups include functional descriptions of 25 specific COG types, with Metabolism being the most prevalent (Fig. 3A; Table S8).

KEGG annotations revealed that protein functions were grouped into six major metabolic systems. Approximately 77.52% of the annotated genes were involved in metabolic functions (Fig. 3A), particularly those related to carbohydrate and amino acid metabolism (Fig. 3B). These results were consistent with the COG data, reinforcing the conclusion that soil microbes predominantly perform metabolic roles. The abundance of carbohydrate- and amino acid-related pathways is likely due to the soil environment’s characteristics, such as abundant humus, plant root exudates, and microbial residues (Abdelrahman et al., 2016; Chantigny, Olk & Angers, 2025). These characteristics provide ample substrates for these metabolic processes and drive soil microorganisms to adapt to such conditions.

To further analyze genes related to carbohydrate metabolism, we used the CAZy database (Fig. 3A). A total of 127,544 CAZyme-encoding genes were identified, classified into seven CAZyme categories. Glycosyl transferases (GT) were the most abundant, followed by glycoside hydrolases (GH) and carbohydrate esterases (CE). These genes were unevenly distributed across MAGs. Notably, GH and GT were especially enriched in Actinomycetota and Pseudomonadota (Fig. 3C). Among all MAGs, MAG975 (*g*_*Actinocrispum*; *s*_*Unclassifiedand*) contained the highest number of functionally annotated genes across all three databases: 6,860 in COG, 5,775 in KEGG, and 517 in CAZy (Table S9).

To assess functional differences among region-specific MAGs, we analyzed the top 10 relatively abundant unique MAGs from each province. Genes related to cell wall/membrane/envelope biogenesis and amino acid transport/metabolism were predominant in MAGs from Sichuan, while MAGs from Guizhou, Yunnan, and Jiangsu were mainly enriched in genes associated with general function prediction, signal transduction, and amino acid metabolism (Fig. S4; Table S8). At the genus level, KEGG analysis showed that *UBA4720* (Sichuan), *Ferruginibacter* (Guizhou), *Sphingomonas_I* (Yunnan), and *Sphingomicrobium* (Jiangsu) had relatively high proportions of annotated genes. Functional pathways common across these genera included global and overview maps, carbohydrate metabolism, and amino acid metabolism (Fig. S5A; Table S1). Similarly, CAZy analysis indicated that genera such as *UBA4720*, *Ferruginibacter*, *Sphingomonas*, and *Flavisolibacter* had relatively high proportions of CAZyme-encoding genes (Fig. S5B).

### Antibiotic resistance gene profiling

**Figure 4:**
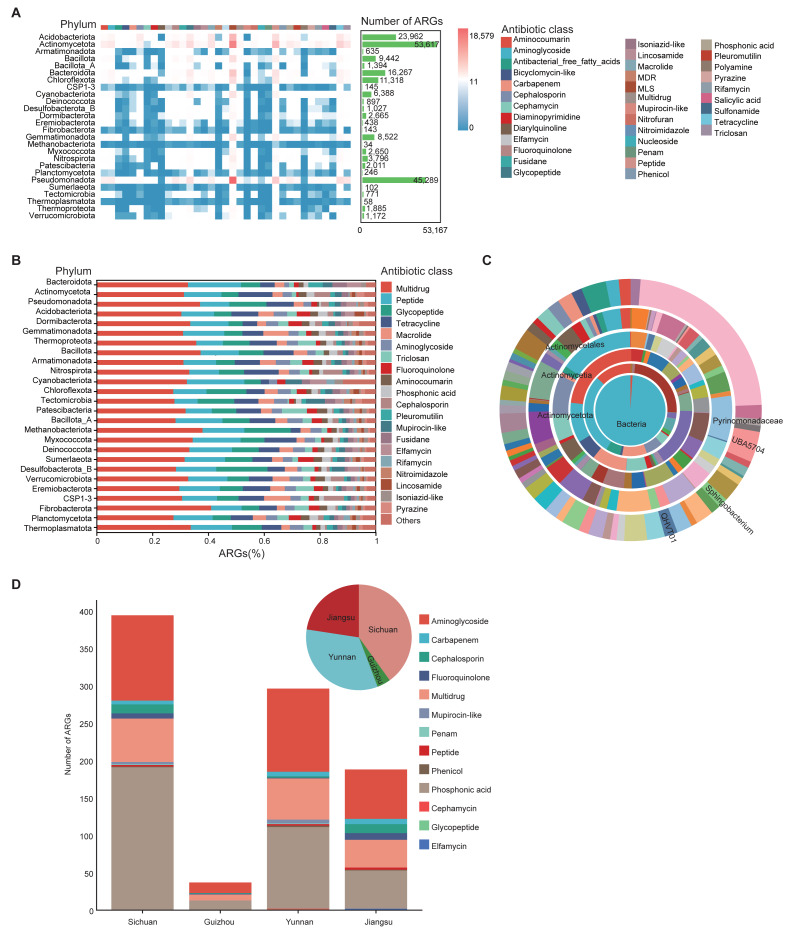
Characterization of ARGs in soil MAGs. (A) Heatmap showing the distribution of ARGs across phyla. Color ranging from blue to red indicates a rise in ARG count; the top color band shows ARG classes, and the green side bars indicate the number of ARGs per phylum. (B) Bar chart summarizing ARG prediction and classification. The *x*-axis shows the proportion of each ARG class; the *y*-axis shows different microbial phyla. Different colored blocks in the columns correspond to different Antibiotic class categories. (C) Concentric diagram showing ARG abundance across taxonomic levels from kingdom (center) to genus (outermost ring). Colors represent distinct species. (D) Distribution of resistance gene counts in province-specific MAGs from Sichuan, Yunnan, Guizhou, and Jiangsu. Bar chart shows gene counts by class (*x*-axis: province; *y*-axis: count). Pie chart represents the proportional composition of resistance gene types by province. Colors represent different Antibiotic classes.

To investigate antibiotic resistance in the soil microbiome, ARGs in 1,136 MAGs from 111 soil samples were annotated using the CARD database. A total of 35 drug resistance classes, comprising 162 distinct ARG types, were identified (Table S10). Among all phyla, Actinomycetota and Pseudomonadota harbored the greatest numbers of ARGs. ARGs were also detected in archaeal phyla such as Thermoproteota and Methanobacteriota (Fig. 4A). Among these, the phylum Methanobacteriota was found to carry 34 ARGs, encompassing 14 distinct ARG classes (Table S10). Multidrug, peptide, and glycopeptide ARGs were the most widely distributed in the soil environment (Fig. 4B).

Genera *QHVT01*, *Sphingobacterium*, and *UBA5704* contained the greatest number of ARGs per genome among taxa represented by at least five MAGs (Fig. 4C), suggesting their potential as ARG reservoirs. The widespread presence of ARGs raises concerns about environmental dissemination through food chains or soil-water-plant systems, potentially threatening ecological and human health.

MAG975 (*g_Actinocrispum*; *s_Unclassified*) and MAG635 (*g_JADGHX01*; *s_Unclassified*), found only in Yunnan Province, harbored the highest number of ARGs according to CARD predictions (Table S9), indicating possible high resistance potential and unique ecological adaptability. All 1,136 MAGs contained at least five ARGs (Table S10), further underscoring the pervasive nature of resistance genes in soil microbial communities.

Of particular note, one *Escherichia coli* strain (MAG817) was identified with 81 distinct ARG types spanning 27 drug resistance classes (Table S11). These included resistance to multidrug, peptide, glycopeptide, tetracycline, macrolide, aminoglycoside, and fluoroquinolone antibiotics. Given the pathogenic nature of *E. coli,* this strain could potentially be a drug-resistant superbug.

ARGs were also observed in several probiotic species, including *Arthrobacter oxydans* (MAG82, MAG174, MAG670, MAG845), *Glutamicibacter arilaitensis* (MAG79, MAG161, MAG537, MAG825), *Pseudomonas_E helleri* (MAG467), *Priestia megaterium* (MAG750), and *Lactobacillus reuteri* (MAG783) (Table S10).

We also compared ARG abundance among soil-specific MAGs from the four provinces. Sichuan, Yunnan, and Jiangsu soils contained significantly more ARGs than Guizhou, with aminoglycoside and phosphonic acid resistance genes being dominant across all regions. In terms of ARG diversity, Sichuan had twice as many antibiotic classes (10) as Guizhou (5), while Yunnan exhibited the greatest diversity with 12 types. Jiangsu followed closely with nine types (Fig. 4D).

### Mobile genetic elements

MGEs are instrumental in the horizontal transmission of ARGs among microbial cells. Understanding their distribution and the relationship between MGEs and ARGs is essential for assessing antibiotic resistance dynamics. A total of 46,662 MGE-related genes were identified across the 1,136 MAGs by aligning gene catalog protein sequences with the MGE Database. These genes were grouped into 68 distinct MGEs and further classified into five types: transposase, integrase, recombinase, conjugative transfer protein, and transposon. Among these, transposase genes were the most abundant, suggesting their major contribution to the dissemination of ARGs (Table S12).

**Figure 5:**
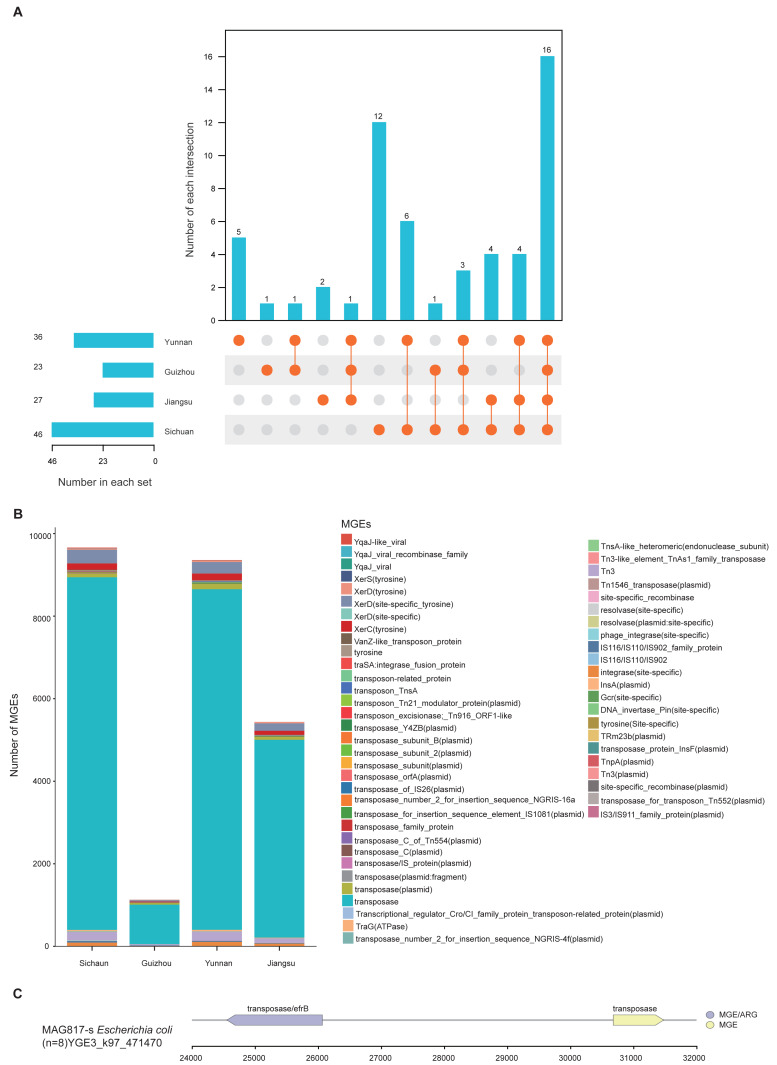
Distribution and mobility potential of MGEs in soil MAGs. (A) Profile and Venn diagram of MGE subtypes identified in province-specific MAGs from Sichuan, Guizhou, Yunnan, and Jiangsu. (B) Comparison of the number and types of MGE subtypes across provinces. (C) Genomic map of gene mobility MAG817 (*E. coli*), highlighting transposase-ARG co-localization with contigs <5 kb.

MGEs can carry and promote the spread of ARGs through horizontal gene transfer (HGT). Thus, the presence and distribution of ARGs and MGEs can serve as indicators of the degree of antibiotic resistance contamination. To explore regional mechanisms of ARG transmission, we analyzed MGE content within the unique MAGs from the four provinces. Results revealed clear regional differences: Sichuan demonstrated the highest diversity (46), followed by Yunnan (36), Jiangsu (27), and Guizhou (23). Regarding province-specific MGE types, Sichuan had 12, Yunnan 5, Guizhou 2, and Jiangsu 1 (Fig. 5A). Among all regions, transposase, XerD (site-specific tyrosine), and Tn3 were the dominant classes (Fig. 5B). Further analysis of gene mobility across the 1,136 MAGs showed that among contig regions shorter than 5 kb, ARGs most frequently appeared adjacent to transposase genes. The genome of MAG975 (*g_Actinocrispum*; *s_Unclassified*) contained the highest number of such co-located regions. Among these, the *bcrA* gene (*n* = 13) was most commonly associated with transposase genes within the same contigs. This suggests a likely mechanism for the horizontal transfer of *bcrA* mediated by transposases in this genome (Table S12).

Previous studies have highlighted *E. coli* as a potential pathogen across various environments. To evaluate the risk of ARG transfer at the strain level, we examined the distribution of MGEs in the genome of MAG817 (*E. coli*). Transposase genes were located adjacent to multiple ARGs within contigs shorter than five kb. For example, a transposase gene was closely linked to the *efrB* gene (Fig. 5C). Given that MAG817 (*E. coli*) was detected in eight out of 111 samples and has a relatively high abundance, this *E. coli* strain may represent a heightened risk for ARG transmission through MGE-mediated horizontal transfer.

**Figure 6:**
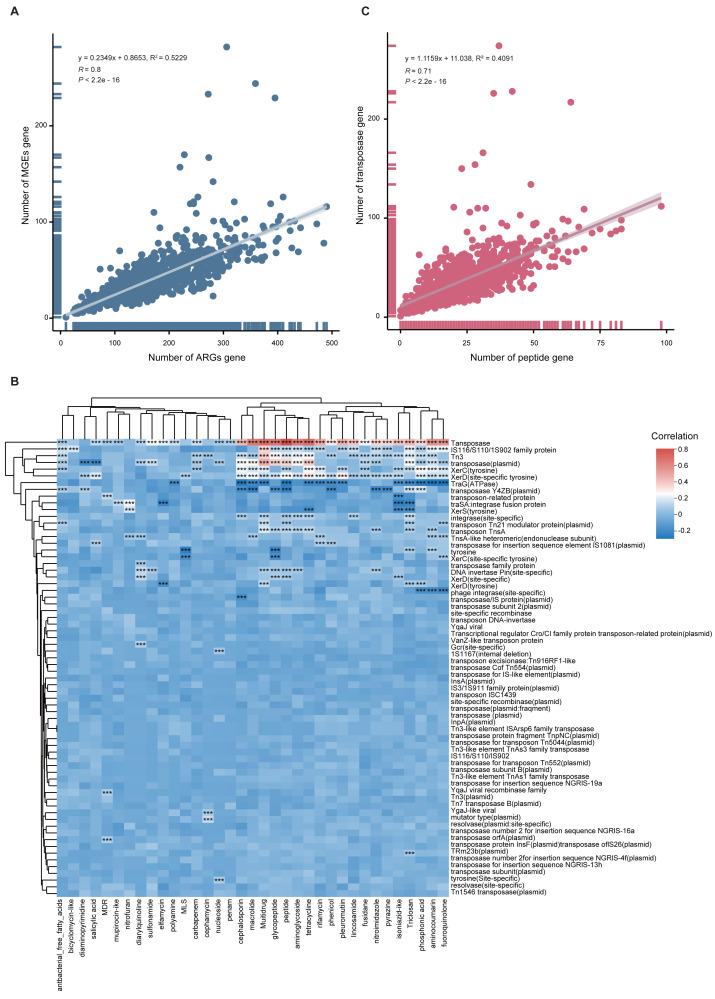
Correlation between ARGs and MGEs. (A) Scatter plot illustrating the linear correlation between total ARG and MGE abundance across 1,136 MAGs. (B) Heatmap showing correlation coefficients between MGE types and ARG classes. Significance is indicated by: *p < 0.05; **p < 0.01; ***p < 0.001. (C) Scatterplot showing strong positive correlation between peptide ARGs and transposase MGEs.

To quantify the relationship between MGEs and ARGs, we performed Spearman and linear regression analyses. A significant positive correlation was observed (*y* = 0.2349x + 0.8653, *R*^2^ = 0.5229, *p* < 0.001), indicating that higher MGE abundance may facilitate ARG proliferation (Fig. 6A). This correlation was also supported by a heatmap (Fig. 6B). Notably, when delving into the individual components of the mobilome, we found a more complex relationship. While transposase genes exhibited a strong positive correlation with peptide ARGs (Fig. 6C), intriguingly, the abundance of *TraG* (a key ATPase gene essential for bacterial conjugation) was negatively correlated with the total ARG abundance (Fig. 6B).

## Discussion

In this study, we constructed a gene catalog for soil microorganisms through metagenome assembly, resulting in 1,136 MAGs. Unlike previous efforts, our sampling encompassed geographically diverse regions across China, including Sichuan, Yunnan, Guizhou, and Jiangsu Provinces. All samples were collected from local soils to reflect different regional characteristics. The recovered MAGs met the medium-quality genome standards defined by Bowers et al. (2017), with ≥ 50% completeness and <10% contamination. This dataset expands the existing soil genome catalogs (Ma et al., 2023; Nayfach et al., 2021), providing a valuable reference for studying uncultured microbiota in complex soil environments.

Taxonomic analysis revealed that the MAGs spanned 23 bacterial and three archaeal phyla, with Actinomycetota and Pseudomonadota being the predominant bacterial phyla, consistent with earlier soil microbiome studies (Cao et al., 2023). Research has found that Actinomycetota often dominate agricultural soils (Yang et al., 2025), and their high abundance is closely related to the degradation process of soil organic matter (Zhang et al., 2019). The results from Alattas et al. (2024) indicate that *Pseudomonas* exhibits strong adaptability in agricultural soils, which is consistent with our findings. Additionally, our study revealed an archaeal relative abundance of 7.12%. According to reports by Cao et al. (2012) the relative abundance of archaea in Chinese soils typically ranges from 0.20% to 9.26%, indicating that the results of this study fall within the typical range. However, compared to the global average archaeal relative abundance in soils, which is approximately 2% (Bates et al., 2011), the value observed in this study is slightly higher. This difference may be attributed to the local warm and humid climate as well as active material cycling conditions (Starke et al., 2021).

There are significant differences in species composition among provinces. Compared to Sichuan, the MAG composition of Yunnan, Guizhou, and Jiangsu showed greater genus-level similarity. Still, each province contained distinct dominant genera—such as *VAYN01*, *UBA4720*, and *JACDBZ01* in Sichuan; *o_Gloeomargaritales*; *g_Unclassified* in Guizhou; *Sphingomonas_I*, *40CM-4-68-19*, and *CADCTB01* in Yunnan; and *Sphingomicrobium* and *VAYN01* in Jiangsu—suggesting strong regional ecological differentiation. Notably, MAG442 *(Lactobacillus amylovorus*) is considerably enriched in samples from Yunnan Province. As a lactic acid bacterium commonly found in carbohydrate-rich environments (Stefanovic, Fitzgerald & McAuliffe, 2017), this region-specific enrichment may be associated with the environmental conditions of Yunnan Province, such as climate, soil type, and vegetation cover. The province’s landforms are mainly characterized by plateaus, basins, hills, and mid- to low-altitude mountains, with an overall tilt from northeast to southwest. The sampling sites had an average elevation of 2,244.00 m, which is significantly higher than the average of 666.61 m in other provinces, indicating a generally high-altitude and rugged terrain. The region experiences a low-latitude plateau monsoon climate, with an annual mean temperature of 15 °C, an average relative humidity of about 64%, and annual precipitation ranging from 634.6 to 1,060 mm, most of which occurs between June and September. Consequently, these differences are likely driven by local environmental factors, soil chemical properties, and the niche-specific adaptations of microorganisms (Bomfim et al., 2025; Thammanu et al., 2021).

Soil microbial communities are essential for maintaining ecosystem stability and resilience (He et al., 2025; Peng et al., 2025; Wagg et al., 2019; Wu et al., 2021). However, the vast majority remain uncultured, limiting our understanding of their metabolic and ecological functions. Metagenomic approaches allow for in-depth exploration of microbial functional potential. Functional annotation using the COG and KEGG databases indicated that metabolism is the primary function of these communities, particularly pathways related to carbohydrate and amino acid metabolism. Amino acid metabolism involves the breakdown of proteins into absorbable units (Miska, Fetterer & Wong, 2014), while carbohydrate metabolism plays a role in energy production and nutrient cycling. Soil organic matter, especially polysaccharide-rich humus, may contribute to the prominence of these pathways. CAZy annotations further highlighted genes involved in GT and GH activity, with some contributions from CE and PL. The enrichment of GT and GH indicates a significant enhancement of both carbohydrate synthesis capacity (function of GT) and degradation capacity (function of GH) by microorganisms in the soil of the study area. This phenomenon aligns with the central role of these enzymes in the local soil carbon cycle. Studies by Jiménez, Chaves-Moreno & Van Elsas (2015) and Storlazzi, Takesue & Hendrix (2025) have demonstrated that GT and GH dominate the carbohydrate-active enzyme spectrum in forest soils, while research by Salam (2018) has shown their predominant role in the carbohydrate-active enzyme profiles of agricultural soils, which is consistent with the conclusions of our study. In addition, at the genus level, the high abundance of CAZyme-encoding genes in *Sphingomonas* is consistent with their known polysaccharide metabolism functions (White, Sutton & Ringelberg, 1996). In contrast, the significant CAZy potential observed in *Ferruginibacter*, *Flavisolibacter*, and *UBA4720* is a relatively new finding.

The prevalence of ARGs in soil microorganisms has become an important research focus (Liu et al., 2024; Willms et al., 2020). This study investigated the distribution of ARGs in 111 soil samples from Yunnan, Guizhou, Sichuan, and Jiangsu. These findings are consistent with previous reports showing ARG persistence in environments lacking direct antibiotic exposure (Fu et al., 2025; Willms et al., 2020). The widespread occurrence of ARGs suggests long-term environmental reservoirs and potential natural origins (Ondon et al., 2021). Pseudomonadota and Actinomycetota were identified as the primary ARG hosts, in agreement with other reports (Mamo, Abera & Tafesse, 2024; Zheng et al., 2022). Notably, ARGs were also detected in *E. coli* and in probiotics such as *Arthrobacter oxydan* and *Glutamicibacter arilaitensis*, suggesting both pathogenic and commensal bacteria may act as resistance reservoirs. Furthermore, Methanobacteriota harbors representatives of nearly every major category of ARGs. The presence of ARGs in Methanobacteriota is likely the result of a combination of natural evolutionary processes, specific environmental conditions, and selective pressures from diverse sources (Evariste, 2023). However, it is still uncertain whether natural environmental conditions primarily drive the formation of these ARGs or whether external pressures play a dominant role. Therefore, future studies should place greater emphasis on elucidating the evolutionary pathways of ARGs in Methanobacteriota and other archaea to comprehensively understand the ecological roles of these genes across diverse environments.

In addition, we have found that the ARG profiles of soils from different regions show significant differences, which are closely related to land use, pollution levels, and the composition of soil microbial communities (Deng et al., 2025; Yang et al., 2021). Soils in Sichuan, Yunnan, and Jiangsu have higher ARG diversity, possibly due to more intensive agricultural activities and antibiotic use (McKinney et al., 2018). Soils in Guizhou have fewer types of ARGs, possibly due to lower human interference and agricultural pollution (Wu et al., 2023). Furthermore, analysis of the relationship between environmental factors and ARG abundance revealed a positive correlation between higher elevation and the prevalence of certain ARG subtypes, such as Mupirocin-like and Phenicol resistance genes. This pattern may be attributed to cooler temperatures and limited human activity in high-altitude areas, which could influence the survival and proliferation of ARG-harboring microorganisms (Zhang et al., 2025).

HGT facilitated by MGEs, including conjugative plasmids, integrative conjugation elements, integrons, and transposons, is a primary mechanism driving the spread of antibiotic resistance (Heuer, Schmitt & Smalla, 2011). We detected diverse MGEs across all soil samples, with transposases being the most abundant. Transposases are key enzymes that mediate the movement of transposons, and their essential function is to facilitate the transfer of adjacent genetic fragments, including ARGs (Qian & Adhya, 2017). Some studies have mentioned that in soil metagenomic data, the abundance of ARGs is significantly positively correlated with the abundance of transposase genes, which can serve as indirect evidence of the transposition element-mediated spread of ARGs (Li et al., 2022), consistent with our research results. This observed dominance of transposases is biologically intuitive, as they are fundamental drivers of gene mobility. Interestingly, we observed a significant negative correlation between TraG, a TraG-like coupling protein associated with the type IV secretion system (T4SS) and known to bind nucleotides/ATP (Schröder & Lanka, 2003; Wallden, Rivera-Calzada & Waksman, 2010; Zechner, Lang & Schildbach, 2012) and ARGs. TraG-family proteins function within conjugation-type T4SSs that mediate plasmid transfer and horizontal gene flow in microbial communities (Gordils-Valentin et al., 2024; Wallden, Rivera-Calzada & Waksman, 2010; Zechner, Lang & Schildbach, 2012). However, the inverse relationship with ARGs suggests that the presence of TraG-associated secretion machinery may not necessarily coincide with the enrichment of resistance genes. One possible explanation is that TraG-containing systems can be linked to mobile elements or plasmids carrying functions other than antibiotic resistance (for example, diverse metabolic traits), reflecting distinct selective pressures (Palomino et al., 2023; Smalla, Jechalke & Top, 2015). Alternatively, the negative correlation may indicate a trade-off between maintaining conjugative machinery and the accumulation of ARGs, where the energetic burden of building/operating conjugation systems and the broader fitness costs of plasmid carriage limit their co-occurrence (Hall et al., 2021; Kusumawardhani et al., 2022; Rajer & Sandegren, 2022; San Millan & MacLean, 2017). This suggests that the contribution of different HGT mechanisms to the resistome may vary under specific environmental conditions. While further experimental validation is required, this observation highlights the complex ecology of secretion systems and resistance gene distribution. Regional variation in MGE type diversity was evident, suggesting local environmental pressures (*e.g.*, land use, heavy metals, or farming practices) may influence HGT dynamics. For example, MAG975 (*g_Actinocrispum*; *s_Unclassified*), the *bcrA* gene frequently co-occurred with transposases, indicating a potential HGT hotspot. Meanwhile, *E. coli* (MAG817) contained multiple ARGs adjacent to MGEs within short contigs, suggesting high mobility potential. Despite being found in only eight samples, its abundance and genomic organization indicate it could pose environmental or public health risks.

## Conclusions

This study systematically analyzed the diversity, distribution characteristics and transmission mechanisms of ARGs in soil microbial communities in four provinces of China. The results showed that soil microorganisms have rich metabolic functions and regionally specific resistance genes and MGEs. Pseudomonadota and Actinomycetota were the main hosts of ARGs, among which strains such as *E. coli* carried multiple resistance genes, suggesting that they may play a key role in the transmission of ARGs. In addition, the detection of MGEs in all samples further confirmed their mediating function in the horizontal transfer of ARGs. There were significant regional differences in the microbial community structure and ARGs profiles among samples from different provinces. These results provide important evidence for understanding the distribution patterns, host characteristics and transmission mechanisms of ARGs in the soil environment, and also provide scientific support for formulating targeted strategies for the prevention and control of drug resistance. Future research can further combine soil physical and chemical properties and land use patterns and other environmental variables to deeply analyze the driving mechanisms of the formation and transmission of ARGs.

##  Supplemental Information

10.7717/peerj.20348/supp-1 Supplemental Information 1 Genomic features and phylogenetic diversity of 1,136 MAGs (A) Assembly statistics for 1,136 MAGs, including total length, contig count, CDS count, GC content, N50, tRNA, rRNAs, and repeat elements. (B) Phylogenetic tree of 1,136 MAGs constructed using GTDB.The legend is ordered by the number of MAGs identiûed in each phylum, from highest to lowest.

10.7717/peerj.20348/supp-2 Supplemental Information 2 Phylum-level overview of shared MAGs among the four provinces

10.7717/peerj.20348/supp-3 Supplemental Information 3 Heatmap of the top 10 most abundant shared MAGs in Sichuan, Guizhou, Yunnan, and Jiangsu

10.7717/peerj.20348/supp-4 Supplemental Information 4 COG Classification Statistical Heat Map The horizontal axis represents different MAGs genomes, and the vertical axis represents the number of genes for different Functions. For the functional descriptions of specific COG types, please refer to the legend below.

10.7717/peerj.20348/supp-5 Supplemental Information 5 A bar chart showing the proportion of KEGG and CAZy functional annotation genes in the top 10 relatively abundant unique MAGs from different regional soil microbial communities (A) The proportion of genes annotated in the KEGG database. (B) The proportion of genes annotated in the CAZy database.

10.7717/peerj.20348/supp-6 Supplemental Information 6 Metagenome - Assembled Genomes data summary of 111 samples

10.7717/peerj.20348/supp-7 Supplemental Information 7 Binning result evaluation table

10.7717/peerj.20348/supp-8 Supplemental Information 8 Genomic statistics results

10.7717/peerj.20348/supp-9 Supplemental Information 9 Statistics of rRNA prediction results

10.7717/peerj.20348/supp-10 Supplemental Information 10 Repeated sequence prediction results statistics

10.7717/peerj.20348/supp-11 Supplemental Information 11 MAGs relative abundance

10.7717/peerj.20348/supp-12 Supplemental Information 12 Relative Abundances of MAGs in Guizhou Province

10.7717/peerj.20348/supp-13 Supplemental Information 13 Statistical table of COG annotation results

10.7717/peerj.20348/supp-14 Supplemental Information 14 Summary of gene function annotations

10.7717/peerj.20348/supp-15 Supplemental Information 15 Resistance gene prediction classification statistics

10.7717/peerj.20348/supp-16 Supplemental Information 16 Statistics on the number of drug-resistant genes in Escherichia coli (MAGS817)

10.7717/peerj.20348/supp-17 Supplemental Information 17 Mobile Genetic Elements Classification Statistics Table

## Additional Information and Declarations

### Data Availability

The following information was supplied regarding data availability: The data is available at NCBI: PRJNA1026177; SRR26329162 to SRR26329272.
