# Supplementary figures and images for: Insights into antibiotic resistomes from metagenome-assembled genomes and gene catalogs of soil microbiota across environments
Source: PeerJ. 2025 Nov 19;13:e20348. doi: 10.7717/peerj.20348 (PMC12640132; doi:10.7717/peerj.20348)

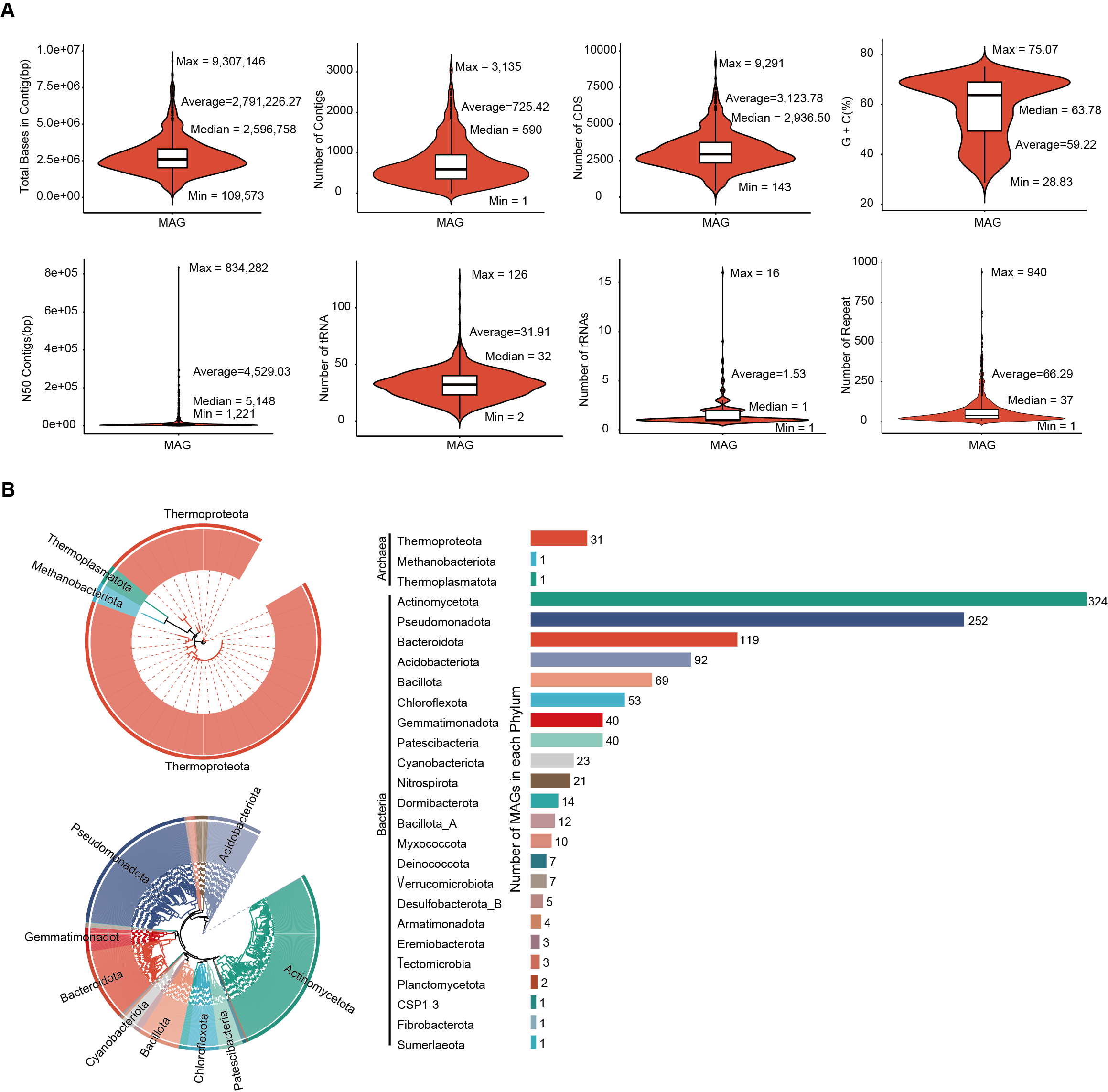

Supplement: Supplemental Information 1 — (A) Assembly statistics for 1,136 MAGs, including total length, contig count, CDS count, GC content, N50, tRNA, rRNAs, and repeat elements. (B) Phylogenetic tree of 1,136 MAGs constructed using GTDB.The legend is ordered by the number of MAGs identiûed in each phylum, from highest to lowest. [file peerj-13-20348-s001.png]

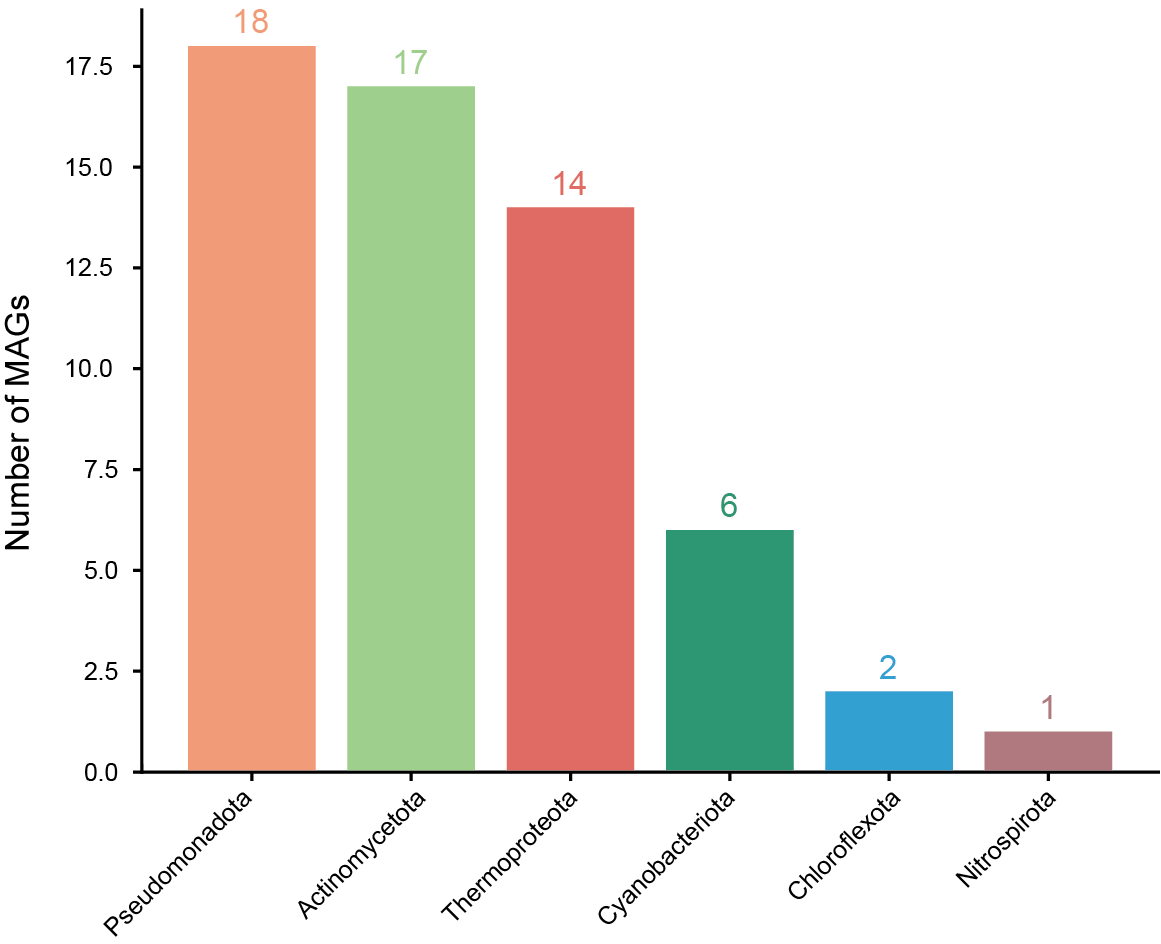

Supplement: Supplemental Information 2 [file peerj-13-20348-s002.png]

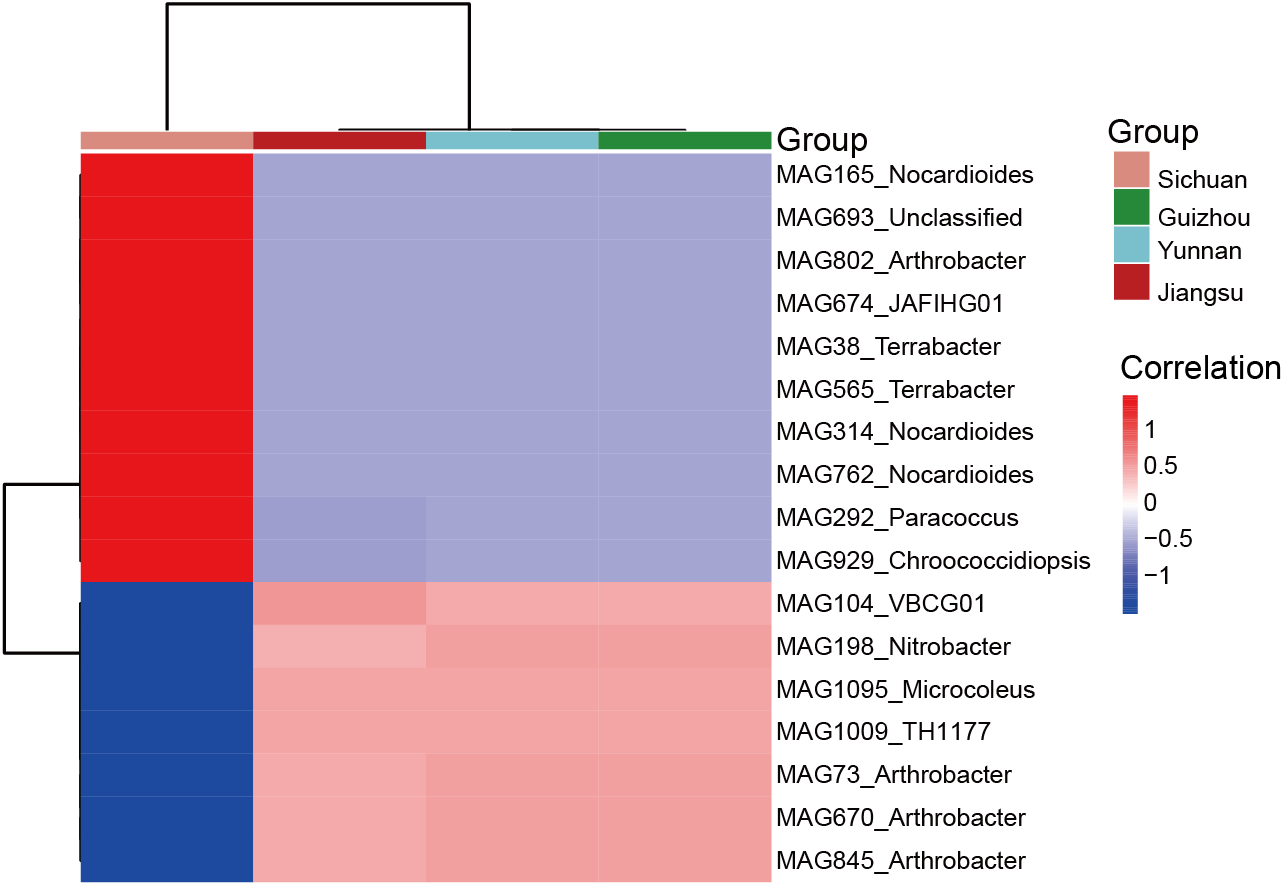

Supplement: Supplemental Information 3 [file peerj-13-20348-s003.png]

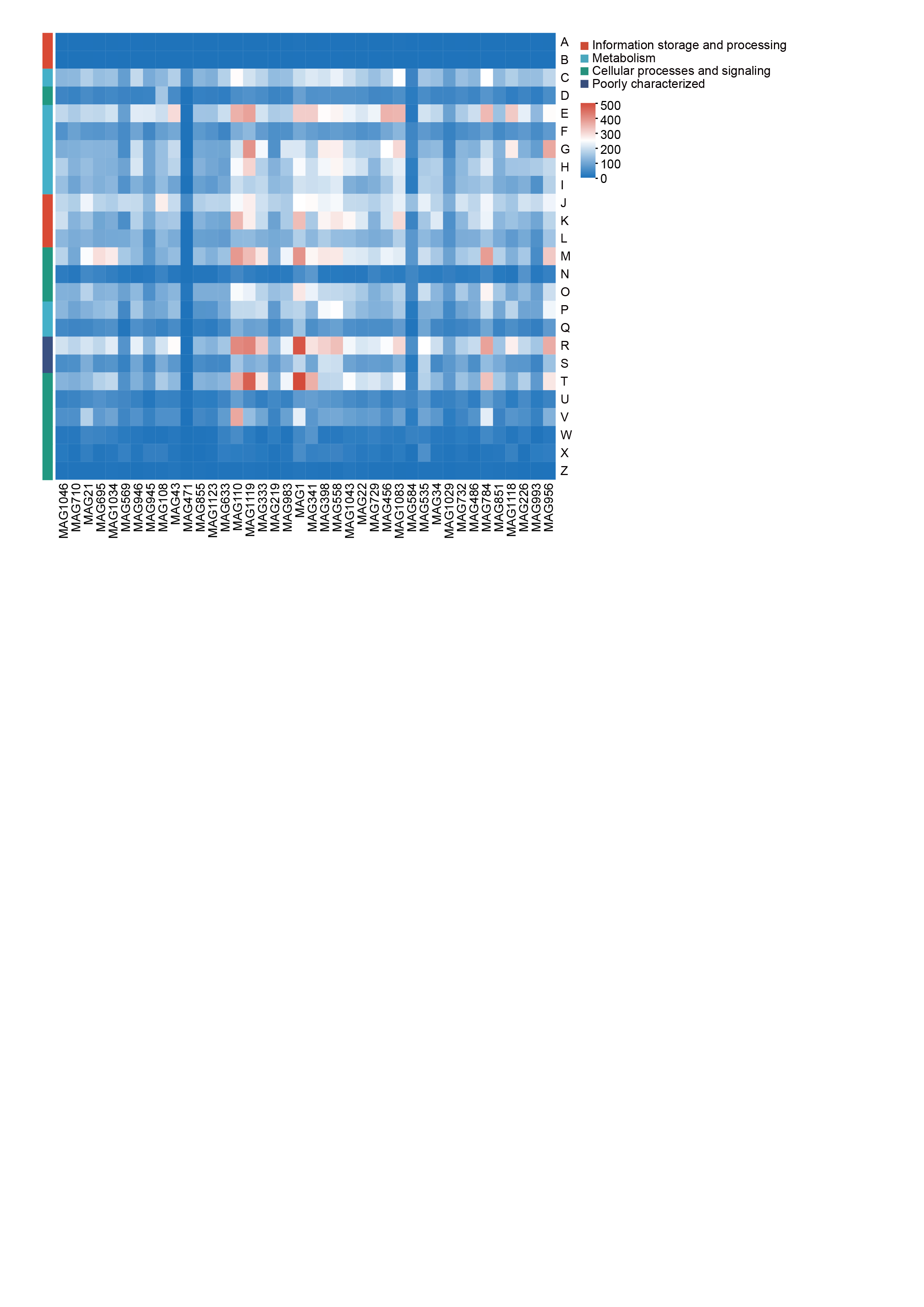

Supplement: Supplemental Information 4 — The horizontal axis represents different MAGs genomes, and the vertical axis represents the number of genes for different Functions. For the functional descriptions of specific COG types, please refer to the legend below. [file peerj-13-20348-s004.png]

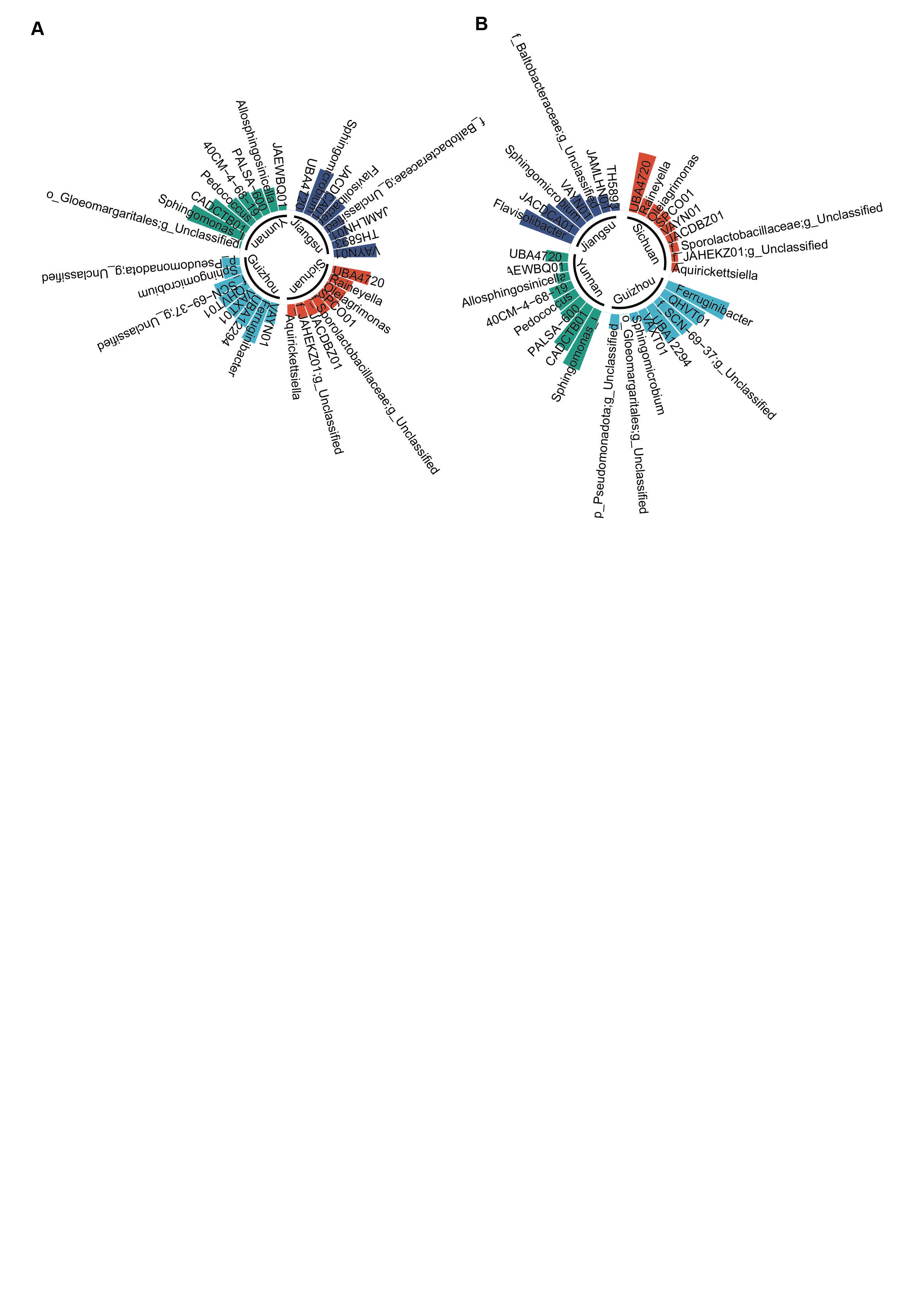

Supplement: Supplemental Information 5 — (A) The proportion of genes annotated in the KEGG database. (B) The proportion of genes annotated in the CAZy database. [file peerj-13-20348-s005.png]
